# A Randomised Controlled Trial of Two Infusion Rates to Decrease Reactions to Antivenom

**DOI:** 10.1371/journal.pone.0038739

**Published:** 2012-06-18

**Authors:** Geoffrey K. Isbister, Seyed Shahmy, Fahim Mohamed, Chandana Abeysinghe, Harendra Karunathilake, Ariaranee Ariaratnam

**Affiliations:** 1 Discipline of Clinical Pharmacology, University of Newcastle, Newcastle, New South Wales, Australia; 2 South Asian Clinical Toxicology Research Collaboration, Peradeniya, Central Province, Sri Lanka; 3 Department of Medicine, Polonnaruwa General Hospital, North Central Province, Polonnaruwa, Sri Lanka; 4 Department of Clinical Medicine, Faculty of Medicine, University of Colombo, Colombo, Sri Lanka; World Health Organization, Switzerland

## Abstract

**Background:**

Snake envenoming is a major clinical problem in Sri Lanka, with an estimated 40,000 bites annually. Antivenom is only available from India and there is a high rate of systemic hypersensitivity reactions. This study aimed to investigate whether the rate of infusion of antivenom reduced the frequency of severe systemic hypersensitivity reactions.

**Methods and Findings:**

This was a randomized comparison trial of two infusion rates of antivenom for treatment of non-pregnant adult patients (>14 y) with snake envenoming in Sri Lanka. Snake identification was by patient or hospital examination of dead snakes when available and confirmed by enzyme-immunoassay for Russell’s viper envenoming. Patients were blindly allocated in a 11 randomisation schedule to receive antivenom either as a 20 minute infusion (rapid) or a two hour infusion (slow). The primary outcome was the proportion with severe systemic hypersensitivity reactions (grade 3 by Brown grading system) within 4 hours of commencement of antivenom. Secondary outcomes included the proportion with mild/moderate hypersensitivity reactions and repeat antivenom doses. Of 1004 patients with suspected snakebites, 247 patients received antivenom. 49 patients were excluded or not recruited leaving 104 patients allocated to the rapid antivenom infusion and 94 to the slow antivenom infusion. The median actual duration of antivenom infusion in the rapid group was 20 min (Interquartile range[IQR]:20–25 min) versus 120 min (IQR:75–120 min) in the slow group. There was no difference in severe systemic hypersensitivity reactions between those given rapid and slow infusions (32% vs. 35%; difference 3%; 95%CI:−10% to +17%;p = 0.65). The frequency of mild/moderate reactions was also similar. Similar numbers of patients in each arm received further doses of antivenom (30/104 vs. 23/94).

**Conclusions:**

A slower infusion rate would not reduce the rate of severe systemic hypersensitivity reactions from current high rates. More effort should be put into developing better quality antivenoms.

**Trial Registration:**

www.slctr.lk SLCTR/2007/005

## Introduction

**Figure 1:**
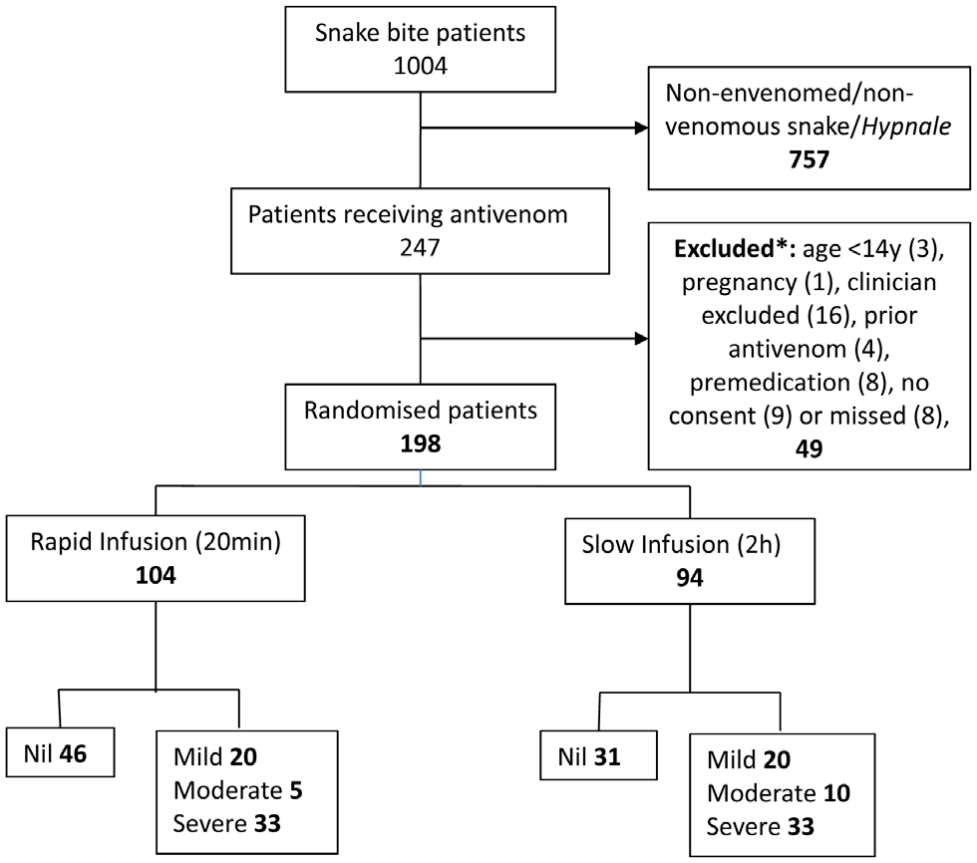
Consort diagram showing all patient recruitments, exclusions and outcomes in each of the arms of the study. * Exclusion criteria were age <14 years, pregnancy, clinician excluded, premedication given, prior administration of antivenom, were missed or did not consent to the study.

Snake envenoming is an important public health problem in tropical and subtropical countries in Africa, Asia, Oceania and Latin America. A recent study estimated that there are at least 440,000 snake envenomings and 20,000 snakebite deaths annually, and potentially as high as five times these figures. [1] Most of the estimated burden of snakebite is from South and Southeast Asia, Sub-Saharan Africa, and Central and South America. This means that large amounts of antivenom are administered in the treatment of snake envenoming in some of these countries where antivenom is available. However, antivenom is made from foreign protein (most commonly equine sources) and is associated with systemic hypersensitivity reactions in a proportion of cases. [2] Antivenom reactions appear to be non-IgE mediated (“anaphylactoid”) but otherwise resemble type I “immediate hypersensitivity” reactions. This has been a particular problem in Sri Lanka where snake envenoming is common, large amounts of Indian polyvalent antivenom are used and severe systemic hypersensitivity reactions or anaphylaxis have been reported in up to 50% of cases in some studies. [3].

The manufacture of high quality antivenom with low reaction rates is cost prohibitive in most parts of the world, so many antivenoms have reaction rates of 30 to 80%. Even in countries such as Australia where high quality antivenoms are produced, reactions occur in 25% of cases with severe reactions in 5% of administrations. [4] For this reason, there have been attempts for decades to reduce the frequency of adverse reactions to antivenom. Premedication has been the commonest intervention to prevent systemic hypersensitivity reactions with the routine recommendations of antihistamines, hydrocortisone and adrenaline for premedication in some regions. [5] The effectiveness of premedication is controversial but a recent large randomized controlled trial did support the use of subcutaneous adrenaline premedication. [6].

Decreasing the rate of infusion of antivenom has also been suggested as a way to decrease the frequency of adverse reactions and it is well-known that rapid infusions may cause complement mediated reactions. However, there are no studies that have investigated the effect of the rate of antivenom infusion on reaction rates. One study reported the adverse effects following the administration of redback spider antivenom in over 2000 cases. [7] In this report there were 11 cases defined as anaphylactoid reactions (0.54%) and in five of these antivenom was given rapidly, undiluted. [7].

There is therefore no good evidence as to whether the rate of infusion affects the risk of systemic hypersensitivity reactions. It is important to determine this because rapid administration of antivenom is often important in severe envenoming to prevent life threatening effects and complications of envenoming. If slower and diluted infusions of antivenom are proved to be safer then consideration of infusion rate will become an important factor in the treatment of snake envenoming, otherwise rapid infusion would be the more appropriate approach.

The aim of this study was to compare the frequency of reactions following the administration of snake antivenom rapidly over 20 minutes versus slowly over 120 minutes.

## Methods

We undertook a randomized, open label, controlled clinical trial comparing two infusion rates of polyvalent antivenom for snake envenoming in Sri Lanka. The primary outcome was the proportion with severe systemic hypersensitivity reactions (“immediate hypersensitivity”; anaphylaxis) within 4 hours of commencement of antivenom.

### Ethics Statement

The study was approved by the Ethical Review Committee, Faculty of Medicine, University of Colombo and was registered with the Sri Lanka Clinical Trials Registry, SLCTR/2007/005. All patients gave written and informed consent to the study. For patients between 14 and 18 years, written informed consent was obtained from the patient’s parents/guardians as well as from the patients themselves. The protocol for this trial and supporting CONSORT checklist are available as supporting information; see Checklist S1 and Protocol S1.

### Study Patients

Patients were recruited from a secondary referral hospital in Chilaw, Sri Lanka between 21^st^ January 2007 and 31^st^ July 2009. All patients (14 years or older) presenting with a snake bite were identified on admission to hospital. However, only patients 16 years were recruited. If a decision was made to administer antivenom to the patient they were eligible for inclusion and were recruited to the study. Exclusion criteria were age less than 14 years, pregnancy, prior administration of antivenom, administration of premedication or inability to give informed consent. Patients could also be excluded by their treating physician. Patients with confirmed hump-nosed viper bites (*Hypnale* spp.) were not treated with antivenom because the polyvalent antivenom is not raised against this snake genus. [8].

### Treatment Protocol

All snakebite patients had baseline clinical data collected, including symptoms and signs of snake envenoming and a 20 minute whole blood clotting test (20 WBCT). When the decision to administer antivenom was made, the study was explained to the patient in their native language (Sinhala, Tamil, or English) by a clinical research assistant and written consent obtained from the patient. The patient was then randomized to one of two parallel treatment arms by the clinical research assistant. Patients in the first treatment group received 10 vials of snake antivenom in 500 ml of normal saline over 20 minutes. Patients in the second treatment group received 10 vials of snake antivenom in 500 ml of normal saline over 2 hours. Patients were not given any premedication prior to the administration of this first dose of antivenom. Polyvalent antivenom (Bharat Serums and Vaccines Limited, India) was the antivenom available during the study.

Randomisation was done with a computer in blocks of four (eg. AABB, ABAB etc.) by GKI. An excel file was created which was then used by MF to create a list which was used to randomize pieces of paper with the wording “20 minute infusion” or “2 hour infusion”. These were put in sealed opaque sequentially numbered envelopes. The clinical research assistants randomised patients by opening these sequentially number envelopes.

After the commencement of antivenom patients were closely observed for a four hour period and put on a cardiac monitor. For the first hour, heart rate (HR), blood pressure (BP), respiratory rate (RR), oxygen saturation and temperature were measured every 5 minutes and then every hour for the remainder of the four hours. Patients were observed for evidence of a systemic hypersensitivity reaction using the Brown grading system, including auscultation of the chest. Any further treatment including repeated doses of antivenom or treatment of antivenom reactions was directed by the attending physician. Patients were observed for the remainder of their admission for any further clinical features or treatments.

All patients had a 10 mL sample of blood collected prior to antivenom administration (baseline) and thence 5 mL samples collected one, four and twelve hours after antivenom administration and thence once daily until discharge. Blood was collected in serum, citrated and EDTA tubes and immediately centrifuged, aliquoted and frozen at −20° Celsius for 1 to 2 weeks and thence at −80° Celsius until the completion of the study.

### Laboratory Assays

Frozen samples were transported to Australia. Frozen serum was then thawed and used to measure Russell’s viper venom concentrations using a previously described enzyme immunoassay (EIA). [9] In brief, polyclonal antibodies (IgG) to Russell’s viper (*Daboia russelli*) were raised in rabbits as previously described. [10] These were conjugated to biotin and then used in a sandwich EIA with the detecting agent streptavidin-horseradish peroxidase. Frozen citrate specimens were thawed to measure the International normalised ratio (INR).

**Table 1:** Demographic features, snake type and clinical effects for patients randomised to the rapid infusion arm and the slow infusion arm.

	Rapid Infusion n = 104	Slow Infusion n = 94
**Sex (male)**	82 (79%)	68 (72%)
**Age (median, IQR); years**	38 (29 to 48)	40 (28 to 52)
**Snake Type**		
Russell’s Viper	84 (81%)	73 (78%)
Krait	3 (3%)	2 (2%)
Cobra	2 (2%)	1 (1%)
Unknown	16 (15%)	17 (18%)
**Clinical Effects**		
Coagulopathy	99 (95%)	87 (93%)
INR>5	55 (53%)	54 (57%)
Neurotoxicity	47 (45%)	47 (50%)

### Data Collection

Demographic information (sex, age), details of the bite (bite site, circumstances of the bite), information on snake identification if available, clinical features of snake envenoming, 20 WBCT results, treatments (antivenom) and outcomes were recorded on datasheets which were then entered into a relational database (Microsoft Access). Baseline observations were recorded prior to antivenom administration. Blood pressure, heart and respiratory rate, oxygen saturation, and any features of systemic hypersensitivity reactions were then recorded.

Based on the clinical features and coagulation studies (INR), clinical envenoming syndrome(s) were defined in each patient as venom induced consumption coagulopathy, neurotoxicity or systemic symptoms (vomiting, headache, abdominal pain). The type of snake was based on identification of the snake by the patient/clinician which was confirmed by EIA for Russell’s viper. Additional cases of Russell’s viper envenoming were determined by EIA.

All systemic hypersensitivity reactions were recorded and classified according to the grading system of Brown. [11] Pyrogenic reactions including patients with fever and/or shivering without other features of systemic hypersensitivity reactions were not included as antivenom reactions.

### Data Analysis

The primary outcome was the proportion of cases where a severe systemic hypersensitivity reaction occurred within 4 hours of commencement of antivenom, as defined by the Brown grading system as grade 3. [11] A severe systemic hypersensitivity reaction was equivalent to anaphylaxis based on international consensus criteria. [12] Secondary outcomes included the proportion with mild and moderate systemic hypersensitivity reactions (Brown grading 1 and 2), duration of hospital stay and repeat antivenom doses. There were a number of predefined laboratory outcomes (cytokines and chemokines) that are not included here.

The sample size for the study was derived from previous studies where approximately 50% of patients administered antivenom had a severe systemic hypersensitivity reaction (most commonly hypotension). [3] A reduction (absolute) of 20% was considered clinically significant and sufficient to warrant changing to a slower infusion rate. In order to detect whether a change in infusion rate reduces the proportion of severe systemic hypersensitivity reactions from 50% to 30%, with a significance level (alpha) of 5% and a power of 80%, a minimum of 103 patients were required in each arm of the trial (i.e. a total of 206 patients). There were no planned interim analyses.

### Statistical Methods

Continuous variables are summarised as medians and interquartile ranges (IQR) to make interpretation easier and proportions are presented with 95% confidence intervals (CIs). The analysis of the primary outcome was intention to treat and the dichotomous outcomes were compared using Fisher’s exact test with a p value ≤0.05 being regarded as statistically significant. Secondary outcomes were summarised and presented as proportions with 95% CIs or median and IQR.

All analyses and graphs were done with GraphPad Prism version 5.03 for Windows (GraphPad Software, San Diego California USA, www.graphpad.com).

**Table 2:** Secondary outcomes for patients randomised to the rapid infusion arm and the slow infusion arm.

	Rapid Infusionn = 104	Slow Infusionn = 94
**Reaction**	58 (56%)	63 (67%)
Nil	46 (44%)	31 (33%)
Mild	20 (19%)	20 (21%)
Moderate	5 (5%)	10 (11%)
Severe	33 (32%)	33 (35%)
**Additional** **Antivenom**	30 (29%)	23 (24%))
Two vials	24 (23%)	20 (21%)
Three vials	6 (6%)	3 (3%)
**Median Length** **of Stay [IQR]**	2 days (2 to 3 days)	2 days (2 to 3 days)
**Deaths**	4 (4%)	0

**Figure 2:**
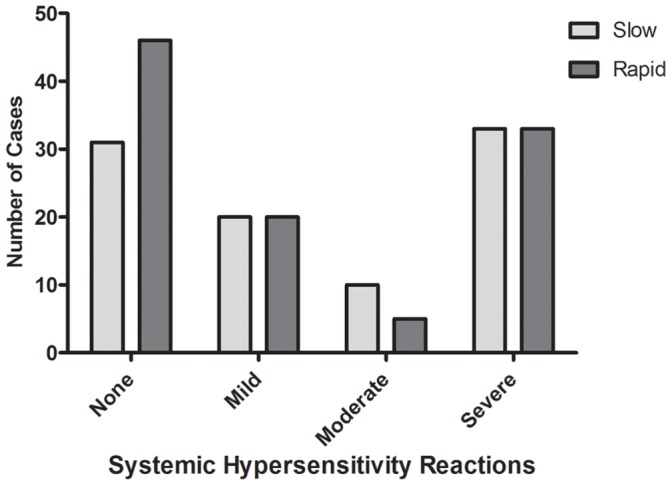
Bar graph showing the number of mild, moderate and severe reactions in each treatment group.

## Results

Of 1004 patients presenting with suspected snakebites during the study period, 247 patients received antivenom. The remaining 757 patients were bitten by non-venomous snakes, were bitten by venomous snakes but not envenomed or bitten by hump-nosed vipers (*Hypnale* spp.). Forty nine patients receiving antivenom were not randomised (see Figure 1) and all 198 patients randomised received antivenom according to their allocation. There were 104 patients allocated to the rapid antivenom infusion and 94 to the slow antivenom infusion (See flowchart - Figure 1).

The two treatment groups had similar baseline characteristics including demographics, type of snake and envenomation syndrome (Table 1). The commonest indication for antivenom was Russell’s viper envenoming with coagulopathy. The median duration of antivenom infusion in the rapid infusion group was 20 minutes (IQR:20 to 25 min) and 120 minutes (IQR:75 to 120 min) in the slow infusion group.

**Table 3:** Comparison of patients with and without antivenom reactions, subdividing into severe and mild/moderate reactions. Proportions are reported with 95% confidence intervals.

	No Reaction (N = 77)	Mild/Moderate Reaction (N = 55)	Severe Reaction (N = 66)
**Age** (median; IQR)	38 y (28 – 45 y)	40 y (27 – 50 y)	42 y (30 – 51 y)
**Sex** (male)	65 (84%; 74 – 91%)	39 (71%; 57 – 82%)	46 (70%; 57 – 80%)
**Snake** (Russell’s viper)	57 (74%; 63–83%)	46 (84%; 71 – 92%)	53 (80%; 68 – 89%)
**Venom Concentration** 1 (median and IQR)	136 (25 – 397 ng/mL)	167 (37 – 461 ng/mL)	150 (46 – 260 ng/mL)
**Clinical Effects**			
Coagulopathy	48 (62%; 51–73%)	35 (64%; 50–76%)	50 (76%; 63–85%)
INR>5	30 (39%; 28–51%)	33 (60%; 46–73%)	29 (44%; 32–57%)
Neurotoxicity	46 (60%; 48–71%)	34 (62%; 48–74%)	36 (55%; 42–57%)

1 Venom concentration for Russell’s viper patients only.

### Primary Outcome

Overall 121 patients (61%) had systemic hypersensitivity reactions to antivenom and 66 (33%) had severe systemic hypersensitivity reactions (anaphylaxis). There were 33 (32%) severe systemic hypersensitivity reactions in the rapid infusion group compared to 33 (35%) in the slow infusion group, with an absolute difference of 3% (95%CI: −10% to +17%), which was not statistically significant (p = 0.65).

### Secondary Outcomes

A comparison of the secondary outcomes is presented in Table 2. There was no difference in the frequency of mild, moderate or severe systemic hypersensitivity reactions between the treatment arms (Table 2 and Figure 2). Similar number of patients in each treatment arm had further antivenom and the length of stay in hospital for each treatment arm was the same.

There were four deaths in the rapid infusion group and none in the slow infusion group. One death occurred following a severe reaction to antivenom with hypotension and hypoxia. One death occurred after transfer to another hospital intensive care unit for hypoxia that developed after the second dose of antivenom. One death occurred in a patient with a mild coagulopathy and acute renal failure with fluid overload from a respiratory arrest who had no reactions to either of two doses of antivenom. The fourth death occurred in a patient with krait envenoming who required intubation and ventilation and died 16 days after admission due to complications of mechanical ventilation.

### Antivenom Reactions

There was no difference in demographics, venom concentration, type of snake or clinical effects between patients not developing reactions and those developing systemic hypersensitivity reactions (Table 3). There were 66 patients with severe systemic hypersensitivity reactions of which 47 (71%) developed hypotension, 32 (48%) developed hypoxia and 13 (20%) developed both hypotension and hypoxia. Thirteen patients developed wheeze, six of these developed hypoxia and seven only had moderate reactions. Shivering was reported in 39 patients which included 13 patients with no other features of immediate hypersensitivity based on the Brown grading system.

**Figure 3:**
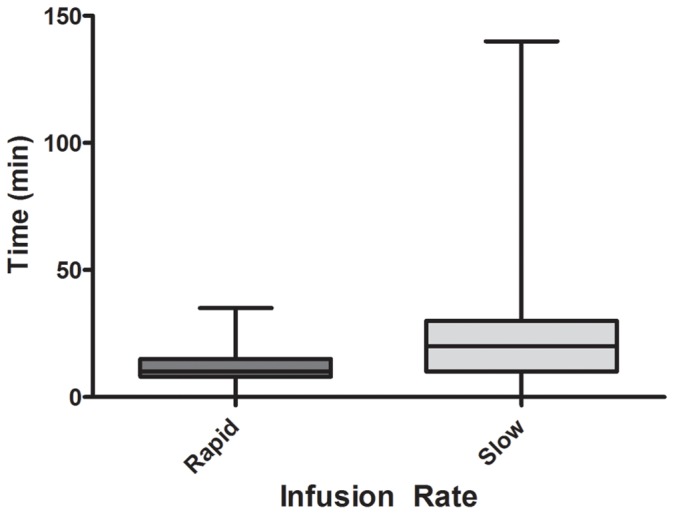
The time of onset of the reaction after antivenom comparing the rapid and slow infusion groups. (The whiskers are the minimum and maximum, the box the interquartile range, and the median line across the box).

The median time of onset of the reaction after antivenom in the rapid infusion group was 10 minutes (IQR: 8 to 15 min; Range: 2 to 35 min) compared to 20 minutes (IQR: 10 to 30 min; Range: 2 to 145 min) in the slow infusion group which was statistically significantly different (p = 0.0002) (Figure 3).

## Discussion

Antivenom reactions were common in this study consistent with previous studies of antivenom in Sri Lanka. [3], [6], [13], [14] A third of patients had severe reactions making this a major problem for clinicians treating snake envenoming. The majority of the severe reactions were of the hypotensive type consistent with previous studies of severe systemic hypersensitivity reactions to antivenom administration. [3], [4] The study suggests that administering 10 vials of antivenom over 20 minutes is as safe as administering 10 vials over 2 hours. The only difference was that the reactions occurred earlier with the rapid infusion. There was no difference between patients who developed reactions and those who did not.

Similar to previous studies of antivenom reactions the majority of severe systemic hypersensitivity reactions were characterised by hypotension. [3], [4] Although hypoxia occurred in just over a third of cases, wheeze was uncommon and hypoxia may have been secondary to hypotension in cases where hypotension co-existed with hypoxia. Recent research suggests there are two types of severe systemic hypersensitivity reactions, hypotensive and hypoxaemic, which is supported by mediator studies where there was significant correlation between IL-6, IL-10, TNF receptor I, mast cell tryptase and histamine concentrations, and hypotensive type anaphylactic reactions, but not with hypoxaemic reactions. [15] Antivenom reactions are of the hypotensive type and appear to be non-IgE mediated (“anaphylactoid”) reactions because previous exposure to antivenom or animal sera is rare.

There are no previous studies comparing infusion rates of antivenom. [5] However there has been a randomised controlled trial comparing the rate of administration of N-acetylcysteine for paracetamol poisoning. [16] The frequency of reactions to N-acetylcysteine was also not reduced by an initial slower infusion rate of 60 minutes compared to 15 minutes. However, similar to our study the reactions occurred later in the 60 minute infusion group. The finding that a slower infusion rate delays but does not prevent reactions with two very different types of medications may have implications for preventive strategies for other agents causing such reactions (e.g. contrast media).

A limitation of the study was that the treating doctors were not blinded to the infusion rate. However, there was adequate concealing of the allocation to each infusion rate using sealed sequentially numbered envelopes. In addition the primary outcome was clearly defined and severe reactions were based on objective criteria including the blood pressure and oxygen saturation.

This study does not support slower infusions of antivenom being used to prevent or reduce the frequency of reactions to antivenom. There was no increase in reactions when infusing antivenom over 20 minutes compared to two hours, and the current infusion time of one hour could be safely reduced to 20 minutes. More rapid infusion would also mean that if reactions occur they will occur earlier and therefore the close observation time for severe reactions following antivenom administration could be shortened.

## Supporting Information

Protocol S1 **Trial Protocol.**
(DOC)

Checklist S1 **CONSORT Checklist.**
(DOC)
