# Supplementary material for: A Randomised Controlled Trial of Two Infusion Rates to Decrease Reactions to Antivenom
Source: PLoS One. 2012 Jun 18;7(6):e38739. doi: 10.1371/journal.pone.0038739 (PMC3377702; doi:10.1371/journal.pone.0038739)
Supplement: Protocol S1 — Trial Protocol. (DOC) [file pone.0038739.s001.doc]

**Projects:** Study on Infusion Rate of Snake Antivenom Therapy

**Background and Justification:**

Snake bite is a common problem in the rural tropics and is responsible for approximately two million snake envenoming worldwide each year (1).This means that large amounts of antivenom are administered in the treatment of snake envenoming. Unfortunately snake antivenom is made from foreign protein

(most commonly equine sources) and are associated with allergic reactions in a proportion of cases. The frequency of allergic reactions depends on the purity and source of the antivenom. It has also been suggested that the rate of infusion effects the frequency or risk of antivenom reactions. . Unfortunately the manufacture of high quality antivenom with low reaction rates is cost prohibitive in many parts of the world, so many antivenoms have reaction rates of 30-80%. The severity and frequency of these reactions has lead to increasing use of premedications to attempt to prevent allergic reactions. There have been routine recommendations of antihistamines, hydrocortisone and adrenaline for premedication (2).

The efficacy of premedication for antivenom administration is controversial with three recent controlled studies having differing results (2-4).It has been suggested that the rate of antivenom infusion may effect the chance of allergic reactions and that rapid infusions may cause complement mediated reactions. There are no studies that have investigated the effect of the rate of antivenom infusion on reaction rates.

There are no published systematic reviews on antivenom infusion rate and the risk of antivenom reactions. It is generally agreed by experts that the rapid administration of antivenom (particularly undiluted) will lead to early complement –mediated reactions (5). One study reported the adverse effects following the administration of redback spider antivenom in over 2000 cases.(6) In this report there were 11 cases defined as ‘anaphylactic’ reactions (0.54%) and in five of theses antivenom was given undiluted and is the likely cause of the reactions.

There is therefore no good evidence as to whether the rate of infusion affects the risk of allergic reactions. It is important to know this because rapid administration of antivenom is often important in severe envenoming to prevent life threatening effects and complications of envenoming. If slower and diluted infusions of antivenom are proved to be safer then consideration of infusion rate will become an important factor in the treatment of snake envenoming, otherwise rapid infusion would be the more appropriate approach if it does not increase the reaction rate.

**References:**

1. White J, Warrell D, Eddleston M, Currie BJ, Whyte IM, Isbister GK. Clinical toxicology – Where are we now? J Toxicol Clin Toxicol 2003; 41(3):263-276.
2. Fan HW, Marcopito LF, Cardoso JL, Franca FO,Malaque CM, Ferrari RA et al. Sequential randomized and double blind trial of promethazine prophylaxis against early anaphylactic reactions to antivenom for bothrops snake bites. BMJ 1999; 318 (7196):1451-1452
3. Premawardhena AP, de Silva CE, Fonseka MMD, Gunathilake SB, de Silva HJ. Low dose subcutaneous adrenaline to prevent acute adverse reactions to antivenom serum in people bitten by snakes: randomized, placebo controlled trial. BMJ 1999; 318: 1041-1043.
4. Currie BJ.Snake bite in tropical Austrailia, Papua New Guinea and Iran Jaya. Emerg Med 2000; 12:285-294.
5. Gawarammana IB, Kularatne SAM, Dissanayake WP, et al. Parallel infusion of hydrocortisone +/- chlopheniramine bolus injection to prevent acute adverse reactions to antivenom for snake bites. *Med J Aust* 2003; 180:20-23.
6. Sutherland SK. Antivenom use in Australia. Premedication, adverse reactions and the use of venom detection kits. Med J Aust 1992;157(11-12):734-739.
7. Muller U, Thurnheer U, Patrizzi R, Spiess J, Hoigne R. Immunptherapy in bee sting hypersensitivity. Bee venom versus wholebody extract. Allergy 1979;34(6):369-378.
8. Theakston RD, Phillips RE, Warrell DA, Galagedera Y, Abeysekara DT, Dissanayaka P et al. Envenoming by the common krait (Bungarus caeruleus) and Sri Lankan Cobra ( Naja naja naja ): efficacy and complications of therapy with Haffkine antivenom. Transactions of the Royal Society of Tropical Medicine & Hygeine 1990; 84 (2):301-308.
9. Eddleston M, Rajapakse S, Rajakanthan, Jayalath S, Sjostrom L, Santharaj W et al. Anti Digoxin Fab fragments in cardiotoxicity induced by ingesion of yellow oleander: randomized controlled trial. Lancet 2000; 355:967-971.
10. Brown SG. Clinical features and severity grading of anaphylaxis. *J Allergy Clin Immunol* 2004;**114:**371-6.
11. Isbister GK, O'Leary MA, Schneider JJ, Brown SG, Currie BJ. Efficacy of antivenom against the procoagulant effect of Australian brown snake (Pseudonaja sp.) venom: In vivo and in vitro studies. Toxicon 2007;49:57-67

**Objectives:**

General Objective

To determine, whether slowing the rate of antivenom infusion will reduce the frequency and severity of antivenom reactions in patients with snake envenoming.

**Research Plan:**

**Study centre**: Base Hospital Chilaw, Anuradhapura, Peradeniya for a period of 12 months. It is an original study, **A randomized, open label, controlled clinical trial.** A total 240 patients will be recruited in this study.

A randomized, double-blind, controlled clinical trial with two parallel groups:

- 10 vials of polyvalent antivenom administered in Normal saline (500ml) over 20 minutes*
- 10 vials of polyvalent antivenom administered in Normal saline (500ml) over 2 hours*

If the patient is administered more than 10 vials then this will be given at the same rate =10 vials over 20 minutes or 2 hours (Although the volume and dilution can be modified for convenience).For example 20 vials given over 40 minutes in 500 ml of Normal saline.

**ii. Detailed description of the research method.**

All patients bitten by a snake will be eligible for the study and will be approached at the time of admission, however they will not formally enter the interventional trial until the decision is made to administer antivenom. All eligible patients will have baseline clinical data collected, including clinical symptoms, signs and a 20 minute whole blood clotting test (20 WBCT). Baseline blood will be collected (see below). When a decision to administer antivenom is made, the patient will be randomized to one of the two parallel treatment arms. Patients in the first treatment group will receive 10 vials of antivenom in 500 ml of normal saline over 20 minutes [alternatively 20 vials of antivenom in 500 ml of normal saline over 40 minutes] Patients in the second treatment group will receive 10 vials of antivenom in 500 ml of normal saline over 2 hrs [alternatively 20 ampoules of antivenom in 500 ml over 4 hours.]

Randomisation will be done by computer in blocks of four (eg. AABB, ABAB etc.) using Microsoft Excel. The computer generated list will be used to randomize pieces of papers to sequentially ordered envelopes with the wording “20 minute infusion” or “2 hour infusion”

Patients will be closely observed for the four hour period following the commencement of administration of antivenom. The patient will be put on a cardiac monitor for this period if possible. For the first hour, HR, BP, RR, oxygen saturation and temperature will be measured every 5 minutes and then every hour for the remainder of the four hours. Patients will be observed for evidence of an allergic reaction using the Brown grading system,(10) including auscultation of the chest. Allergic reactions will be treated as per the current protocol of antivenom reactions with intravenous fluids and 0.5 mg of intramuscular adrenaline into the lateral thigh (or upper arm if not possible).

A 15 mL sample of blood will be taken using a sterile technique (syringe and needle) from each patient.

- 1. The 15mL sample will be divided between one serum (red top tube), one EDTA tube (purple top) and one plasma citrate (blue top) tube.
  2. The serum tube will be spun and the serum aliquoted into 3 portions and frozen.
  3. The citrate plasma tube will be immediately double spun and then aliquoted into two separate tubes and immediately frozen.
  4. The EDTA tube will be spun and aliquoted into two separate containers.

The specific identity of the snake will be determined by ELISA on blood samples and blood concentrations will be determined with the same method retrospectively.(11) Normal coagulation studies will be performed on samples for patients who developed coagulapthy. Creatine kinase will be measured in patients with Russell’s viper bite. Serial mast cell tryptase, complement and anaphylactic mediators will be measured in all patients who receive antivenom. Urine will be collected in patients with Russell’s viper bite. If the snake is brought to the hospital this will be photographed or formally identified and recorded. Blood films will be made where possible and stored for the study.

Throughout the trial, the patient will remain under the responsibility of the admitting consultant. Both study and host hospital clinicians will report to this individual.

**How the sample size was determined:**

In two previous studies approximately 50% of patients administered antivenom had a Brown grade 2 or 3 systemic allergic reaction (most commonly hypotension). (4,8) A reduction (absolute) of 20% would be clinically significant and would be sufficient to warrant the use of premedication. In order to detect whether premedication reduces the rate of allergic reactions from 50% to 30%, with a significant level (alpha) of 5% and a power of 80%, a minimum of 103 patients must be recruited to each arm of the trial (i.e. A total of 206 patients). To ensure appropriate recruitment and loss of patients the aim will be to recruit 240 patients.

**iii. Description of data analysis procedure**

The RCT aims to determine the effectiveness of two different infusion rates of antivenom administration. The primary analysis will be done by intention-to-treat using the chi squared test for the primary outcome (or Fisher’s exact test if appropriate) and for other dichotomous outcomes. The relative risk (and risk reduction)and absolute risk reduction (with 95% confidence intervals) will also be calculated. For the outcomes (mainly the primary outcome) where the time –to-event is recorded, the log rank test will be used to compare treatment groups.

Analysis of trends in treatment effect for factors ‘initial dose of antivenom’ and ‘use of antivenom prior to hospital admission’ will also be performed using statistical modeling techniques.

Admission blood samples will be retrospectively analysed to confirm the identity of the snake. The primary analysis will then be repeated to correct for this.

The main hypothesis is that a slower infusion rate of antivenom (10 vials over 2 hours versus 20 minutes) will reduce the rate of all severe allergic reactions following antivenom administration from 50% to 30%.

We also want to determine if the severity of antivenom reaction is inversely related to the severity of envenoming. This will be done by comparing the concentration of venom in patient’s blood (ELISA) to severity of reaction for individual snake species.

**Subject Selection**

**Participant characteristics:**

Age more than 14 years

Non pregnant

**Explain recruitment procedures:**

Team members will be on 24hrs a day in the study hospitals. They will be notified of any snake bite admissions by the doctor on duty in the OPD or by the medical wards so they can recruit the patients. Chilaw, Anuradhapura, Peradeniya hospitals manage about 500 snake bite patients annually and of these approximately 50% will receive antivenom. Past experience of poisoning trials in Sri Lanka suggest a high recruitment rate (>90% in a trial of anti-digoxin Fab RCT(9)); so we would conservatively estimate 80% patients would be recruited. This means that 200 patients will be recruited over a one year period.

**Inclusion and exclusion criteria.**

Study will recruit all patients that have a history of snake bite and randomize all patients that then receive antivenom. The exclusion criteria will be

- Age < 14 years
- Pregnancy
- Previous administration of antivenom

All appropriate methods have been used to avoid bias:

1. Randomization method is independent of the investigators and dependent on a computer algorithm;
2. Treatment allocation cannot be predicted in advance
3. The primary and secondary outcomes are objective measures
4. All outcomes will be recorded prospectively by the study team and not by the treating doctors
5. Patient follow-up will be complete
6. Analysis will be by intention to treat.

**Description of all medical procedures and safety measures**

All eligible patients will have baseline clinical data collected, including clinical symptoms, signs and a whole blood clotting test (WBCT). Baseline blood will be collected (see below).

Patients in the first treatment group will receive 10 vials of antivenom in 500 ml of normal saline over 20 minutes. Patients in the second treatment group will receive 10 vials of antivenom in 500 ml of normal saline over 2 hrs. Patients will be closely observed for the four hour period following the commencement of administration of antivenom. The patient will be put on a cardiac monitor for this period if possible. For the first hour, HR, BP, RR, oxygen saturation and temperature will be measured every 5 minutes and hourly for the remainder of the four hours. Patients will be observed for evidence of an allergic reaction using the Brown grading system, including auscultation of the chest. Allergic reactions will be treated as per the current protocol of antivenom reactions with intravenous fluids and adrenaline.

A 15 ml sample of blood will be taken using a sterile technique (syringe and needle) from each patient consenting to the study. Normal coagulation studies will be performed on samples for patients who developed coagulopathy. Creatine kinase will be measured in patients with Russell’s viper bite. Serial mast cell tryptase, complement and anaphylactic mediators will be measured in all patients who receive antivenom by the laboratory of Assoc. Prof Simon Brown in Western Australia. Urine will be collected in patients with Russell’s viper bite.

Throughout the trial, the patient will remain under the responsibility of the admitting consultant. Both study and host hospital clinicians will report to this individual.

**Primary outcome:** proportion with severe systemic allergic reactions (grade 3 by Brown grading system) within 4 hours of commencement of antivenom.

**Secondary outcomes:** proportion with elevated mast cell tryptase: proportion with elevated complement; hospital length of stay; proportion with mild and moderate allergic reaction (grade 1 and 2 Brown grading); and proportion with delayed administration of further antivenom therapy. Proportion developing serum sickness will be determined in a smaller number because follow up after discharge is difficult in the rural hospital setting.

Brown Grading System for systemic allergic reactions.

| Grade | Clinical Effects |
| --- | --- |
| **I**  Mild(skin and subcutaneous tissues only) | Generalized erythema,urticaria, periorbital oedema or angioedema. |
| **2**  Moderate(features suggesting respiratory, cardiovascular, or gastrointestinal involvement) | Dysponea,stridor,wheeze,nausea,vomiting,  dizziness (pre syncope) diaphoresis, chest or throat tightness or abdominal pain. |
| **3**  Severe (hypoxia, hypotension or neurological compromise) | Cyanosis or SpO2 less than or equal 92% at any stage, hypotension,(SBP<90mmHg in adults)confusion, collapse, LOC or incontinence. |

**Informed Consent**

Written informed consent will be requested from conscious patients by a study physician in the patient’s own language. For patients between 14 and 16 years, written informed consent will be obtained from the patient’s parents’/guardians; consent will also be sought from these young patients themselves. Consent for unconscious patients will be sought from accompanying relatives. Unaccompanied unconscious patients will not be recruited.

**iv. Ethical consideration:**  ethical approval obtained from the Ethical Review Committee of the Colombo Medical Faculty.
